# High-Fidelity Virtual Objective Structured Clinical Examinations with Standardized Patients in Nursing Students: An Innovative Proposal during the COVID-19 Pandemic

**DOI:** 10.3390/healthcare9030355

**Published:** 2021-03-20

**Authors:** Oscar Arrogante, Eva María López-Torre, Laura Carrión-García, Alberto Polo, Diana Jiménez-Rodríguez

**Affiliations:** 1 Fundación San Juan de Dios, Centro de Ciencias de la Salud San Rafael, Universidad de Nebrija, Paseo de La Habana, 70, 28036 Madrid, Spain; elopezt@nebrija.es (E.M.L.-T.); lcarrion@nebrija.es (L.C.-G.); apolo@nebrija.es (A.P.); 2 Departamento de Enfermería, Fisioterapia y Medicina, Universidad de Almería, 04120 Almería, Spain; d.jimenez@ual.es

**Keywords:** clinical competence, COVID-19, high fidelity simulation training, learning, nursing education, video conferencing, virtual simulation

## Abstract

In response to the cancellation of in-person objective structured clinical examinations (OSCEs) prompted by confinement due to the COVID-19 pandemic, we designed a solution to adapt our traditional OSCEs to this new reality in nursing education. We implemented an innovative teaching proposal based on high-fidelity virtual OSCEs with standardized patients. The purposes of our study were to describe this innovative teaching proposal and compare nursing competence acquisition in final year nursing students through virtual and in-person OSCE modalities. The study included 234 undergraduate students: 123 students were assessed through high-fidelity virtual OSCEs during May 2020, whereas 111 students were assessed through in-person OSCEs during May 2019. The structure of OSCEs, including its stations, clinical simulated scenarios, and checklists, was the same in both OSCE modalities. The effect size of the differences among the competence categories of checklists, including their total scores, was small. Regarding our virtual OSCEs was similarly successful to in-person OSCEs, this online format was found to be useful, feasible, and cost-saving when in-person OSCE was not possible. Therefore, high-fidelity virtual OSCEs with standardized patients could be considered as another choice of OSCE not only in the current COVID-19 pandemic but could also be extended to normal situations, even post-pandemic.

## 1. Introduction

Clinical simulation methodology has increased exponentially over the last few years and has gained acceptance in nursing education. Simulation-based education is considered an effective educational methodology for nursing students to achieve the competencies needed for their professional future [1,2,3,4]. Additionally, simulation-based educational programs have been demonstrated to be more useful than traditional teaching methodologies [3,5]. As a result, most nursing faculties are integrating this methodology into their study plans [6]. Simulation-based education has the potential to shorten the learning curve for students, increase the fusion between theoretical knowledge and clinical practice, establish deficient areas in students, develop communication and technical skills acquisition, improve patient safety, standardize the curriculum and teaching contents, and offer observations of real-time clinical decision making [4,5,7,8].

Simulation-based education offers an excellent opportunity to perform not only observed competency-based teaching, but also the assessment of these competencies. In this sense, simulated-based assessment is aimed at evaluating various professional skills, including knowledge, technical and clinical skills, communication, and decision-making; as well as higher-order competencies such as patient safety and teamwork skills [1,2,3,9]. Compared with other traditional assessment methods (i.e., written or oral test), simulation-based assessment offers the opportunity to evaluate the actual performance in an environment similar to the ‘real’ clinical practice, assess multidimensional professional competencies, and present standard clinical scenarios to all students [1,2,3,9].

Objective structured clinical examination (OSCE) is commonly conducted in the simulation-based assessment as a summative evaluation to evaluate students’ clinical competence [10]. Summative evaluation is used to establish the learning outcomes achieved by students at the end of the course [11]. This evaluation strategy is helpful to educators in evaluating students’ learning, the competencies acquired by them, and their academic achievement [12]. This assessment is essential in the education process to determine readiness and competence for certification and accreditation [9,13]. Consequently, OSCE has been used by educational institutions as a valid and reliable method of assessment. OSCE most commonly consists of a ‘round-robin’ of multiple short testing stations, in each of which students must demonstrate defined clinical competencies, while educators evaluate their performance according to predetermined criteria using a standardized marking scheme, such as checklists. Students must rotate through these stations where educators assess students’ performance in clinical examination, technical skills, clinical judgment, and decision-making skills during the nursing process [10,14]. This strategy of summative evaluation incorporates actors performing as simulated patients. Therefore, OSCE allows assessing students’ clinical competence in a real-life simulated clinical environment. After simulated scenarios, this evaluation strategy provides educators with an opportunity to give students constructive feedback according to their achieved results in the checklist [9,10,13,14].

However, the outbreak of the novel coronavirus disease 2019 (COVID-19) forced many governments to implement public health measures to reduce the spread and contagion of the virus [15,16,17] in March 2020, including limiting human contact [18]. Thus, the Spanish government declared the state of alarm on 15 March 2020 by the Royal Decree No. 463/2020 [19], confining the entire population, including the closure of universities. Consequently, the confinement due to the COVID-19 pandemic resulted in the suspension of all in-person curricular activities in most universities around the world. Particularly, most planned in-person final OSCEs were canceled. This situation presented significant challenges for nursing education. It is essential to ensure final year students of health sciences are not delayed in their incorporation into the health system as healthcare professionals, especially in times of healthcare crisis. As a result, this pandemic significantly impacted final year students’ preparedness, particularly affecting their transition from student to healthcare professional [20]. Despite its cancellation, this situation has promoted the adaptation of simulation-based education to new digital technologies. In this sense, some universities and clinical simulation centers adapted their in-person OSCEs, implementing virtual OSCEs using online platforms of video conferences, mainly Zoom™ software (Zoom Video Communications Inc., San Jose, CA, USA) [21,22,23,24,25]. However, most of these innovative simulation experiences have been conducted for medical students, so its implementation is needed for nursing students.

In response to the cancellation of in-person OSCEs prompted by the confinement due to the COVID-19 pandemic, we designed a solution to adapt our traditional OSCEs to this new reality in nursing education. We implemented an innovative teaching proposal based on high-fidelity simulation methodology during the COVID-19 confinement, implementing virtual OSCEs with standardized patients. It should be noted that high-fidelity simulation refers to simulation experiences that are extremely realistic and provide a high level of interactivity and realism for the learner [26]. Consequently, we implemented virtual OSCEs following the standards of high-fidelity simulation to increase the fidelity of our virtual proposal.

Therefore, the purposes of our study were to describe this innovative teaching proposal and compare nursing competence acquisition in final year nursing students through virtual and in-person OSCE modalities.

## 2. Materials and Methods

### 2.1. Study Design

A descriptive cross-sectorial study, using a quantitative methodology, was conducted to compare nursing competencies acquisition through virtual and in-person OSCE modalities.

### 2.2. Participants and Setting

The study included 4th-year undergraduate Nursing Degree students within the subject ‘Supervised clinical placements–advanced level’. It should be noted that OSCEs are included within this subject in our university center, and they were carried out when all nursing students had completed all their clinical practices. In no case did OSCEs replace face-to-face clinical practices, not even when these practices were canceled due to the COVID-19 confinement. A total of 234 nursing students participated in the study (123 students were assessed through high-fidelity virtual OSCEs during May 2020, whereas 111 students were assessed through in-person OSCEs during May 2019). The study was carried out at a university center in Madrid (Spain) which teaches Physiotherapy and Nursing Degrees. 

### 2.3. Virtual OSCE Design

When university face-to-face classes were canceled, all OSCE stations originally programmed for the in-person OSCEs were adapted and reformulated to an online format due to the new confinement situation. A total of eight simulated clinical scenarios were designed related to hospitalized patients or treated in primary care. The structure of OSCEs, including its stations, clinical simulated scenarios, and checklists, was the same in both OSCE modalities.

It should be noted that the implementation of virtual OSCEs with standardized patients followed the Standards of Best Practice recommended by the International Nursing Association for Clinical Simulation and Learning (INACSL) [27,28,29,30]. In this way, all the stages included in a high-fidelity session were accomplished: pre-briefing, briefing, simulated scenario, and debriefing. Although traditional OSCEs do not usually include the debriefing phase, we decided to include this phase in all OSCEs carried out in our university center, since we consider this phase is quite relevant to nursing students’ learning process and their imminent professional career. All of these stages were conducted using the platform Blackboard Collaborate Launcher™ (Blackboard Inc., Reston, VA, USA) provided by the university, a virtual platform of online video conferences. 

All nursing students formed work teams of three students in all OSCE stations. To establish a safe psychological learning environment, an online joint conference with all nursing students was carried out 1 week before the performance of OSCE stations. In this pre-briefing phase, we implemented several activities based on practices recommended by the INACSL Standards Committee [27,29] and Rudolph, Raemer, and Simon [31] for establishing a psychologically safe context (Table 1).

Each work team had to perform two OSCE stations: a clinical case of a hospitalized patient and another case of a patient treated in primary care. All these simulated scenarios were randomly assigned to each work team and enough differences were introduced in all simulated scenarios so they were not recognized by the rest of the students. In the briefing phase, brief information related to the simulated scenario was presented to each work team on the screen of their home computers for two minutes before entering each OSCE station. 

During the simulated scenario, a standardized patient appeared on the computer screen and interacted with the students according to the nursing activities they performed. Regarding standardized patients, all of them were trained to play their roles in each OSCE station to ensure a high level of fidelity experience [32]. Although they changed during the different OSCE stations, all standardized patients were selected based on their experience in clinical simulation methodology and performed the role of a patient following the Standards of Best Practice recommended by the Association of Standardized Patient Educators (ASPE) [32]. If the students wished to perform any nursing technique or a medical complimentary test, they were asked to describe how they would perform it and if they performed it correctly, the result (i.e., an electrocardiogram) was shown to them on the computer screen. Similarly, an image of the patient’s ulcer or surgical wound was shown on the computer screen, allowing the nursing students to assess it. If the patient was monitored, a monitor appeared on the computer screen showing continuously the patient’s vital signs in real-time according to his/her clinical situation. In this sense, the free version of the Simpl-Simulated Patient Monitor™ application (available for iOS and Android) was installed on a Tablet and its screen was duplicated on the computer used by the professors of the subject. Furthermore, all nursing students were able to view simultaneously the standardized patient, the monitoring of his/her vital signs, the corresponding result of the medical complimentary test, or the image of his/her wound through the screen sharing option available on the virtual platform used. During the execution of each OSCE station, professors checked if the nursing students performed or not the required nursing activities. All OSCE stations lasted 10 min.

After each OSCE station was concluded, a debriefing was conducted to give students feedback about their performance. The debriefings in each OSCE station lasted 10 min and they were carried out according to the Plus-Delta debriefing tool [33], a technique recommended when time is limited. Within these debriefings, professors communicate to students the total score obtained in the appropriate checklist. Subsequently, the nursing students were directed to another room of video conferences to perform the following OSCE station.

Finally, it should be noted the in-person OSCEs were carried out, as usual, in the clinical simulation laboratories placed in the university center.

### 2.4. Data Collection

The nursing students who were assessed through high-fidelity virtual OSCEs performed them during May 2020, when in-person OSCEs were canceled due to the COVID-19 confinement. In contrast, the nursing students who were assessed through in-person OSCEs performed them during May 2019, before the COVID-19 pandemic. 

Professors assessed nursing students’ clinical performance using checklists (‘Yes’/‘No’), checking if the students performed or not the required nursing activities during the execution of each OSCE station. It should be noted the professors who assessed nursing students were the authors of this manuscript. These checklists for evaluating OSCE stations were based on nursing activities selected by consensus among professors, registered nurses, and clinical placement mentors. All checklists were previously pilot-tested before nursing students were evaluated. It should be noted the same simulated scenarios were performed in both OSCE modalities, applying the same checklists. Nursing activities were divided into five competence categories: nursing assessment, clinical judgment/decision-making, clinical management/nursing care, communication/interpersonal relationships, and teamwork. Table 2 shows the checklist of the required nursing competencies in an OSCE station that recreated a critically ill patient with diagnosis of exacerbation of Chronic Obstructive Pulmonary Disease (COPD).

### 2.5. Data Analysis

Data were analyzed using IBM SPSS Statistics version 24.0 software for Windows (IBM Corp., Armonk, NY, USA). Descriptive statistics were calculated to interpret the results obtained in demographic data and clinical performance during OSCEs. T-test was used to compare nursing competence acquisition through both OSCE modalities. Cohen’s d was calculated to analyze the effect size for *t*-tests. Statistical tests were two-sided (α = 0.05), so the statistical significance was set at 0.05.

### 2.6. Ethical Considerations

The research committee of the university center approved the study (P_2018_012). According to the ethical standards, all participants received written informed consent and written information about the study and its goals. Additionally, written informed consent for audio-video recording was obtained from all participants.

## 3. Results

The age of nursing students ranged from 21 to 43 years (mean = 23.64; SD = 3.35). Most students were women (n = 195; 83.3%).

Descriptive data for each competence category of checklists and its total scores, *t*-test, and effect sizes (d) of differences between high-fidelity OSCEs and in-person OSCEs are shown in Table 3. The students assessed through high-fidelity OSCEs obtained total scores that ranged from 60 to 90 points (mean = 68.13; SD = 17.96), whereas students assessed through in-person OSCEs obtained total scores that ranged from 65 to 95 points (mean = 68.82; SD = 13.96). Furthermore, the competence acquisition evaluated in nursing students was similar using both OSCE modalities, according to the scores obtained in the five competence categories evaluated (nursing assessment, clinical judgment and decision-making; clinical management and nursing care; communication and interpersonal relationships; and teamwork). Therefore, the effect size of the differences among competence categories of checklists, including its total scores, was small (Cohen’s d values > 0.2 and <0.5) [34].

## 4. Discussion

We adapted our OSCEs on an online format to respond to the inability to give face-to-face classes at university due to the confinement by the COVID-19 pandemic. As a result, we designed high-fidelity virtual OSCEs with standardized patients that comply with all the requirements and Standards of Best Practices proposed by the INACLS [27,28,29,30] and the ASPE [32].

Regarding the small effect size of the differences obtained in our study, our innovative teaching proposal based on virtual OSCEs was similarly successful to in-person OSCEs. This result is congruent with previous studies that conducted virtual OSCEs on an online format in medical and dental students [23,24,25]. Although this phase is rarely present in OSCEs, our students recognized the significance of debriefing as shown the most evidence found [1,35,36,37]. The debriefing phase allows nursing students to learn from their mistakes, especially when they are about to enter their professional careers. In this sense, learn from error is one of the most advantages of the clinical simulation shown in several studies [4,5,38] and mistakes should be considered learning opportunities rather than there being embarrassment or punitive consequences [39]. 

The practical utility is considered as another advantage of OSCE and clinical simulation methodology, reducing the gap between theory and practice [4,5,40,41]. Therefore, it helps nursing students to be prepared for their future careers. According to Benner’s model of skill acquisition in nursing [42], nursing students become competent nurses through this learning process, acquiring a degree of safety and clinical experience before their professional careers [43]. In this sense, we consider our students obtained an adequate level of nursing competence acquisition through both OSCE modalities, regarding the means of each competence category and the total score obtained in the checklists applied. 

In contrast, our innovative teaching proposal on online format using a virtual platform was a new activity to assess the clinical performance of nursing students. We assumed this novelty could increase the traditional students’ complaints about OSCE. Reduced time is a frequent complaint of students in OSCE [14,44] and clinical simulation methodology [4,5,9]. In this sense, professors, registered nurses, and clinical placement mentors tested all OSCE stations and their checklists in this study. All of them checked the time was enough for its resolution. Increased anxiety is another criticism of OSCE. Several studies have demonstrated students’ anxiety increase during simulation sessions and may impact negatively their learning process [45,46], so it is considered as the most disadvantage of clinical simulation [1,2,3,4,5,6,7,8,9]. However, the best solution to reduce these complaints is the orientation of students to the simulated environment [9,10,13,14]. Consequently, we implemented several activities based on internationally accepted practices [27,29,31] for establishing a psychologically safe context and orienting nursing students before performing high-fidelity virtual OSCEs with standardized patients. Therefore, we consider it is essential to implement this pre-briefing phase to ensure the effectiveness of the subsequent virtual OSCE since its implementation impacted positively on the overall success of our innovative teaching proposal. 

The main limitation of our study is the inability to properly assess students’ performance of technical skills, such as nursing techniques or medical complementary tests. This limitation has been also indicated in previous virtual OSCEs [21,22,23,24,25]. We mitigated this inherent limitation of this OSCE modality by asking nursing students to describe accurately and in detail the technique or test they would perform. Another limitation of our innovative proposal is technical problems related to the platform of video conferences used, home computers, or internet access. In our study, there were only a few problems related to technical difficulties which were easily solvable. In addition, although the checklists employed in OSCE have been criticized for their subjective construction [9,10,13,14], we constructed them with the expert consensus of nursing professors, registered nurses, and clinical placement mentors. Finally, future studies should compare nursing competence acquisition through both OSCEs modalities in the same group of students. These studies should also analyze the satisfaction of students, standardized patients, and instructors with this methodology, as well as apply high-fidelity virtual OSCEs among different students of health sciences (e.g., Nursing, Medicine, Physiotherapy), and, lastly, expand this methodology to other settings, countries, and education centers.

## 5. Conclusions

Our innovative teaching proposal based on high-fidelity virtual OSCEs with standardized patients is a response to the needs of simulation-based education prompted by the COVID-19 pandemic and its related restrictions. Regarding the overall success of our virtual OSCEs, this online format was found to be useful, feasible, and cost-saving when in-person OSCEs are not possible. Beyond the current restrictions, this online format would allow universities and clinical simulation centers to perform virtual OSCEs while saving on travel time and costs. Therefore, high-fidelity virtual OSCEs with standardized patients could be considered as another choice of OSCE not only in the current COVID-19 pandemic but could also be extended to normal situations, even post-pandemic.

## Figures and Tables

**Table 1:** Activities implemented for establishing a psychologically safe context.

List of Activities
Detailed explanation of development phases of objective structured clinical examinations (OSCE) in virtual modality with standardized patients. Clarifying expectations and resolving the concerns about the procedure of high-fidelity virtual OSCEs through a platform of online video conferences. Checking the computer equipment (camera and microphone), performing a demonstration test. The premise agreed upon: error is a learning opportunity (mistakes are free of risk or consequences). Clarifying the role of the facilitator: honest, flexible, and adaptable. He/she provides constructive feedback and maintains professional integrity. Establishing a “fictional contract” with participants. Confidentiality agreement and commitment to respect students. Creation of operational work teams composed of 3 nursing students. Random assignment of simulated clinical scenarios for each work team. Presentation of OSCE stations: all students received essential information about each OSCE station prior to its performance.

**Table 2:** Checklist of the required nursing competencies in the exacerbation of Chronic Obstructive Pulmonary Disease (COPD) OSCE station.

**Nursing Assessment (20 Points)**	**Yes**	**No**	**Points**
They perform a focused respiratory exploration through appropriate pulmonary auscultation (5 points)			
They recognize correctly signs and symptoms of respiratory distress, including SaO_2_ (5 points)			
They assess correctly hemodynamic signs and symptoms (5 points)			
They interpret correctly the complementary tests ordered by the physician (5 points)			
**Clinical Judgement and Decision-Making (20 Points)**	**Yes**	**No**	**Points**
They diagnose correctly the patient’s clinical situation (5 points)			
They prioritize adequately nursing interventions (5 points)			
They re-evaluate the patient according to nursing assessment (5 points)			
They apply the appropriate treatment for respiratory distress at the right time (5 points)			
**Clinical Management and Nursing Care (30 Points)**	**Yes**	**No**	**Points**
Handwashing (2.5 points)			
Use of gloves (2.5 points)			
They place the patient in semi-Fowler position (2.5 points)			
Proper pulse oximeter placement (2.5 points)			
Proper EEG electrodes placement (2.5 points)			
Proper blood pressure cuff placement (2.5 points)			
They apply correctly the adequate oxygen therapy according to nursing assessment (2.5 points)			
They call a physician (2.5 points)			
They follow properly physician instructions (2.5 points)			
They administer correctly the prescribed medication (2.5 points)			
They evaluate the patient’s response to the medical treatment administered (2.5 points)			
They perform correctly the complementary test ordered by the physician (2.5 points)			
**Communication and Interpersonal Relationships (15 Points)**	**Yes**	**No**	**Points**
They introduce themselves to the patient (3 points)			
They reduce the patient’s anxiety (3 points)			
They show empathy, active listening, and respect when they communicate with the patient and/or family (3 points)			
Appropriate communication with the physician (3 points)			
Appropriate communication among team members (3 points)			
**Teamwork (15 Points)**	**Yes**	**No**	**Points**
Appropriate coordination among team members and they demonstrate an effective teamwork			
	**Total**		

**Table 3:** Descriptive data, *t*-test, and effect sizes (d) of differences between in-person OSCEs and virtual OSCEs for each competence category of checklists and its total scores.

Scale	In-Person OSCEs ^1^	Virtual OSCEs ^1^	F	Sig.	Effect Size (d)
	Mean (SD ^2^)	Mean (SD ^2^)			
Nursing assessment	11.89 (4.31)	11.67 (4.11)	2.35	0.50	0.27
Clinical judgement and decision-making	10.27 (5.39)	9.84 (4.70)	4.11	0.33	0.29
Clinical management and nursing care	21.08 (5.29)	20.88 (5.38)	1.98	0.56	0.26
Communication and interpersonal relationships	12.65 (2.75)	12.13 (2.44)	4.21	0.10	0.32
Teamwork	12.97 (5.20)	12.45 (4.07)	4.03	0.24	0.30
Total score	68.82 (13.96)	68.13 (17.96)	5.14	0.10	0.42

^1^ OSCEs: Objective structured clinical examinations; ^2^ SD: Standard deviation.

## Data Availability

The data presented in this study are available on request from the corresponding author.

## References

[1] Cant R.P., Cooper S.J. (2010). Simulation-based learning in nurse education: Systematic review. J. Adv. Nurs..

[2] Chernikova O., Heitzmann N., Stadler M., Holzberger D., Seidel T., Fischer F. (2020). Simulation-based learning in higher education: A meta-analysis. Rev. Educ. Res..

[3] Kim J., Park J.H., Shin S. (2016). Effectiveness of simulation-based nursing education depending on fidelity: A meta-analysis. BMC Med. Educ..

[4] Ricketts B. (2011). The role of simulation for learning within pre-registration nursing education—A literature review. Nurse Educ. Today.

[5] Shin S., Park J.H., Kim J.H. (2015). Effectiveness of patient simulation in nursing education: Meta-analysis. Nurse Educ. Today.

[6] Bagnasco A., Pagnucci N., Tolotti A., Rosa F., Torre G., Sasso L. (2014). The role of simulation in developing communication and gestural skills in medical students. BMC Med. Educ..

[7] Oh P.J., Jeon K.D., Koh M.S. (2015). The effects of simulation-based learning using standardized patients in nursing students: A meta-analysis. Nurse Educ. Today.

[8] Stayt L.C., Merriman C., Ricketts B., Morton S., Simpson T. (2015). Recognizing and managing a deteriorating patient: A randomized controlled trial investigating the effectiveness of clinical simulation in improving clinical performance in undergraduate nursing students. J. Adv. Nurs..

[9] Ryall T., Judd B.K., Gordon C.J. (2016). Simulation-based assessments in health professional education: A systematic review. J. Multidiscip. Healthc..

[10] Harden R.M., Gleeson F.A. (1979). Assessment of clinical competence using an objective structured clinical examination (OSCE). Med. Educ..

[11] Billings D.M., Halstead J.A. (2012). Teaching in Nursing: A Guide for Faculty.

[12] Nichols P.D., Meyers J.L., Burling K.S. (2009). A framework for evaluating and planning assessments intended to improve student achievement. Educ. Meas. Issues Pract..

[13] Oermann M.H., Kardong-Edgren S., Rizzolo M.A. (2016). Summative simulated-based assessment in nursing programs. J. Nurs. Educ..

[14] Mitchell M.L., Henderson A., Groves M., Dalton M., Nulty D. (2009). The objective structured clinical examination (OSCE): Optimising its value in the undergraduate nursing curriculum. Nurse Educ. Today.

[15] Wang C., Horby P.W., Hayden F.G., Gao G.F. (2020). A novel coronavirus outbreak of global health concern. Lancet.

[16] Lotfi M., Hamblin M.R., Rezaei N. (2020). COVID-19: Transmission, prevention, and potential therapeutic opportunities. Clin. Chim. Acta.

[17] Nagesh S., Chakraborty S. (2020). Saving the frontline health workforce amidst the COVID-19 crisis: Challenges and recommendations. J. Glob. Health.

[18] Vellingiri B., Jayaramayya K., Iyer M., Narayanasamy A., Govindasamy V., Giridharan B., Ganesan S., Venugopal A., Venkatesan D., Ganesan H. (2020). COVID-19: A promising cure for the global panic. Sci. Total Environ..

[19] Spanish Government (2020). Real Decreto 463/2020, de 14 de Marzo, por el que se Declara el Estado de Alarma Para la Gestión de la Situación de Crisis Sanitaria Ocasionada por el COVID-19.

[20] Choi B., Jegatheeswaran L., Minocha A., Alhilani M., Nakhoul M., Mutengesa E. (2020). The impact of the COVID-19 pandemic on final year medical students in the United Kingdom: A national survey. BMC Med. Educ..

[21] Boursicot K., Kemp S., Ong T., Wijaya L., Goh S.H., Freeman K., Curran I. (2020). Conducting a high-stakes OSCE in a COVID-19 environment. MedEdPublish.

[22] Boyle J.G., Colquhoun I., Noonan Z., McDowall S., Walters M.R., Leach J.P. (2020). Viva la VOSCE?. BMC Med. Educ..

[23] Hannon P., Lappe K., Griffin C., Roussel D., Colbert-Getz J. (2020). An objective structured clinical examination: From examination room to Zoom breakout room. Med. Educ..

[24] Kakadia R., Chen E., Ohyama H. (2020). Implementing an online OSCE during the COVID-19 pandemic. J. Dent. Educ..

[25] Lara S., Foster C.W., Hawks M., Montgomery M. (2020). Remote assessment of clinical skills during COVID-19: A virtual, high-stakes, summative pediatric objective structured clinical examination. Acad. Pediatr..

[26] Lioce L., Lopreiato J., Downing D., Chang T.P., Robertson J.M., Anderson M., Diaz D.A., Spain A.E., The Terminology and Concepts Working Group (2020). Healthcare Simulation Dictionary.

[27] INACSL Standards Committee (2016). INACSL standards of best practice: Simulation^SM^ simulation design. Clin. Simul. Nurs..

[28] INACSL Standards Committee (2016). INACSL standards of best practice: Simulation^SM^ facilitation. Clin. Simul. Nurs..

[29] INACSL Standards Committee (2016). INACSL standards of best practice: Simulation^SM^ simulation glossary. Clin. Simul. Nurs..

[30] INACSL Standards Committee (2016). INACSL standards of best practice: Simulation^SM^ Debriefing. Clin. Simul. Nurs..

[31] Rudolph J.W., Raemer D., Simon R. (2014). Establishing a safe container for learning in simulation: The role of the presimulation briefing. Simul. Healthc..

[32] Lewis K.L., Bohnert C.A., Gammon W.L., Hölzer H., Lyman L., Smith C., Thompson T.M., Wallace A., Gliva-McConvey G. (2017). The Association of Standardized Patient Educators (ASPE) Standards of Best Practice (SOBP). Adv. Simul..

[33] Decker S., Fey M., Sideras S., Caballero S., Rockstraw L., Boese T., Franklin A.E., Gloe D., Lioce L., Sando C.R. (2013). Standards of Best Practice: Simulation Standard VI: The Debriefing Process. Clin. Simul. Nurs..

[34] Cohen L., Manion L., Morrison K. (2011). Research Methods in Education.

[35] Dufrene C., Young A. (2014). Successful debriefing-best methods to achieve positive learning outcomes: A literature review. Nurse Educ. Today.

[36] Levett-Jones T., Lapkin S. (2014). A systematic review of the effectiveness of simulation debriefing in health professional education. Nurse Educ. Today.

[37] Neill M.A., Wotton K. (2011). High-fidelity simulation debriefing in nursing education: A literature review. Clin. Simul. Nurs..

[38] King A., Holder M.G., Ahmed R.A. (2013). Error as allies: Error management training in health professions education. BMJ Qual. Saf..

[39] Higgins M., Ishimaru A., Holcombe R., Fowler A. (2012). Examining organizational learning in schools: The role of psychological safety, experimentation, and leadership that reinforces learning. J. Educ. Chang..

[40] Hope A., Garside J., Prescott S. (2011). Rethinking theory and practice: Pre-registration student nurses experiences of simulation teaching and learning in the acquisition of clinical skills in preparation for practice. Nurse Educ. Today.

[41] Lisko S.A., O’Dell V. (2010). Integration of theory and practice: Experiential learning theory and nursing education. Nurs. Educ. Perspect..

[42] Benner P. (1984). From Novice to Expert: Excellence and Power in Clinical Nursing Practice.

[43] Nickless L.J. (2011). The use of simulation to address the acute care skills deficit in pre-registration nursing students: A clinical skill perspective. Nurse Educ. Pract..

[44] Kelly M.A., Mitchell M.L., Henderson A., Jeffrey C.A., Groves M., Nulty D.D., Glover P., Knight S. (2016). OSCE best practice guidelines-applicability for nursing simulations. Adv. Simul..

[45] Cantrell M.L., Meyer S.L., Mosack V. (2017). Effects of simulation on nursing student stress: An integrative review. J. Nurs. Educ..

[46] Nielsen B., Harder N. (2013). Causes of student anxiety during simulation: What the literature says. Clin. Simul. Nurs..

